# Ticagrelor Inhibits Toll-Like and Protease-Activated Receptor Mediated Platelet Activation in Acute Coronary Syndromes

**DOI:** 10.1007/s10557-019-06932-7

**Published:** 2020-02-15

**Authors:** Patricia P. Wadowski, Constantin Weikert, Joseph Pultar, Silvia Lee, Beate Eichelberger, Renate Koppensteiner, Irene M. Lang, Simon Panzer, Thomas Gremmel

**Affiliations:** 1 Department of Internal Medicine II, Medical University of Vienna, Vienna, Austria; 2 Department of Blood Group Serology and Transfusion Medicine, Medical University of Vienna, Vienna, Austria; 3 Department of Internal Medicine, Cardiology and Nephrology, Landesklinikum Wiener Neustadt, Wiener Neustadt, Austria

**Keywords:** Toll-like receptors, Protease-activated receptors, Antiplatelet therapy, P2Y_12_ antagonists, Cardiovascular disease

## Abstract

**Purpose:**

Since ticagrelor inhibits the cellular uptake of adenosine, thereby increasing extracellular adenosine concentration and biological activity, we hypothesized that ticagrelor has adenosine-dependent antiplatelet properties. In the current study, we compared the effects of ticagrelor and prasugrel on platelet activation in acute coronary syndrome (ACS).

**Methods:**

Platelet surface expression of P-selectin and activated glycoprotein (GP) IIb/IIIa in response to adenosine diphosphate (ADP), the toll-like receptor (TLR)-1/2 agonist Pam3CSK4, the TLR-4 agonist lipopolysaccharide (LPS), the protease-activated receptor (PAR)-1 agonist SFLLRN, and the PAR-4 agonist AYPGKF were measured by flow cytometry in blood from 80 ticagrelor- and 80 prasugrel-treated ACS patients on day 3 after percutaneous coronary intervention. Residual platelet aggregation to arachidonic acid (AA) and ADP were assessed by multiple electrode aggregometry and light transmission aggregometry.

**Results:**

ADP-induced platelet activation and aggregation, and AA-induced platelet aggregation were similar in patients on ticagrelor and prasugrel, respectively (all *p* ≥ 0.3). Further, LPS-induced platelet surface expression of P-selectin and activated GPIIb/IIIa did not differ significantly between ticagrelor- and prasugrel-treated patients (both *p* > 0.4). In contrast, Pam3CSK4-induced platelet surface expression of P-selectin and activated GPIIb/IIIa were significantly lower in ticagrelor-treated patients (both *p* ≤ 0.005). Moreover, SFLLRN-induced platelet surface expression of P-selectin and activated GPIIb/IIIa were significantly less pronounced in patients on ticagrelor therapy compared to prasugrel-treated patients (both *p* < 0.03). Finally, PAR-4 mediated platelet activation as assessed by platelet surface expression of activated GPIIb/IIIa following stimulation with AYPGKF was significantly lower in patients receiving ticagrelor (*p* = 0.02).

**Conclusion:**

Ticagrelor inhibits TLR-1/2 and PAR mediated platelet activation in ACS patients more strongly than prasugrel.

## Introduction

Patients suffering acute coronary syndromes (ACS) are routinely treated with dual antiplatelet therapy (DAPT) consisting of aspirin and an adenosine diphosphate (ADP) P2Y_12_ antagonist for 12 months to prevent detrimental platelet activation and subsequent atherothrombotic events [1]. According to the guidelines of the American College of Cardiology/American Heart Association and the European Society of Cardiology [2, 3], the newer P2Y_12_ receptor antagonists ticagrelor and prasugrel should be preferred over clopidogrel in ACS patients undergoing percutaneous coronary intervention (PCI) with stent implantation due to their more favorable effects on 1-year ischemic outcomes in large randomized clinical trials [4, 5]. The latter are most likely due to faster, stronger, and more consistent inhibition of ADP-induced platelet activation by ticagrelor and prasugrel compared to clopidogrel [4–10]. Indeed, previous studies revealed a similar inhibitory effect of ticagrelor and prasugrel on residual ADP-induced platelet aggregation [11, 12]. However, human platelets can be activated by a myriad of agonists via different receptors [7, 13]. Among these are toll-like receptors (TLR) and protease-activated receptors (PAR) which are not targeted by standard DAPT but might contribute to platelet hyperreactivity as well as to the development and progression of atherosclerotic cardiovascular disease [14–16].

TLR are part of innate immunity recognizing pathogen-associated molecular patterns of microbial origin and damage-associated molecular patterns of injured host cells [17, 18]. However, the role of TLR extends far beyond: TLR in the myocardium are involved in pro-inflammatory cytokine expression, infarct size regulation, and influence cardiac remodeling [14, 17, 19]. In addition, TLR seem to be involved in the modulation of ischemia-reperfusion injury in ACS [20]. TLR-1, TLR-2, TLR-3, TLR-4, TLR-6, TLR-7, and TLR-9 were found on and in human platelets, respectively [14], and may represent a link between thrombosis and inflammation [16]. Acute myocardial infarction leads to the upregulation of platelet TLR-1 and TLR-4 [21, 22]. Moreover, it has been shown that platelets from ACS patients on DAPT can be directly and dose-dependently activated by Pam3CSK4, a synthetic agonist of TLR-1/2, whereas platelet activation via TLR-4 in these patients occurred only in response to the highest concentration of lipopolysaccharide (LPS) [22]. Further, the TLR-2/6 agonist FSL-1 was not able to induce platelet activation at any concentration in ACS patients [22]. Hence, in particular, the TLR-1/2 pathway appears to be functional in ACS and may contribute to platelet activation despite state-of-the-art DAPT. The response of human platelets to thrombin is partially mediated by the autocrine/paracrine effect of secreted ADP after thrombin-induced platelet activation [23]. Accordingly, in vitro experiments in blood from healthy volunteers showed that inhibition of the P2Y_12_ but not P2Y_1_ receptor reduced thrombin-induced platelet activation [24]. In line with these findings, Whitley et al. described an effect of ticagrelor on PAR-4 mediated platelet aggregation [25]. Moreover, as shown by Kälvegren et al., TLR-1/2 mediated platelet activation can at least partially be affected by ADP receptor antagonists [26]. Further, platelet inhibition by ticagrelor has been associated with less in vitro platelet-monocyte complex formation and a modulation of TLR-2- and TLR-4 mediated cytokine response of peripheral blood mononuclear cells [27].

PAR-1 and PAR-4 enable human platelet activation by the serine protease thrombin, one of the strongest platelet agonists [7, 28, 29]. While PAR-1 is sensitive to low levels of thrombin, PAR-4 triggers platelet activation and aggregation only at high thrombin concentrations, and cleavage of PAR-4 by thrombin occurs 20- to 70-fold slower than cleavage of PAR-1 [30]. Recently, we have shown that platelet responsiveness to PAR-1 and PAR-4 stimulation is preserved in the majority of clopidogrel-treated patients and in about 20% of patients receiving prasugrel following angioplasty with stent implantation [31, 32].

Due to their distinct molecular structures, the cyclopentyl-triazolo-pyrimidine ticagrelor and the thienopyridine prasugrel may differently affect platelet activation which may in turn explain clinical differences between ticagrelor and prasugrel in large randomized trials [4, 5, 8, 9, 33]. Since ticagrelor inhibits the cellular uptake of adenosine, thereby increasing extracellular adenosine concentration and biological activity [34, 35], we hypothesized that ticagrelor has adenosine-dependent antiplatelet properties [36]. In the current study, we therefore compared the effects of ticagrelor and prasugrel on platelet activation in 160 ACS patients undergoing PCI and stenting. Platelet surface expression of P-selectin and activated GPIIb/IIIa in response to ADP, Pam3CSK4, LPS, SFLLRN, and AYPGKF were determined to assess agonist-induced platelet activation. ADP was chosen as agonist to document the response to platelet inhibition therapy, as all patients received an ADP P2Y_12_ antagonist. Platelet response to Pam3CSK4 was measured because it has been shown previously that, in particular, the TLR-1/2 pathway is functional in ACS in the presence of DAPT [22]. LPS-induced platelet surface expression of P-selectin and activated GPIIb/IIIa was assessed to investigate platelet activation via TLR-4 [37]. SFLLRN- and AYPGKF-induced platelet activation was determined to evaluate the platelet response to PAR-1 and PAR-4 stimulation, respectively [38, 39].

## Materials and Methods

### Study Population

The study had an observational, non-randomized open label design. The study population consisted of 160 ACS patients on daily aspirin (100 mg/day), and either prasugrel (10 mg/day, *n* = 80), or ticagrelor (180 mg/day, *n* = 80) therapy. All patients were Caucasians from the Vienna urban area and were recruited within 2 years for the study. Blood sampling was performed on day 3 after acute successful PCI with stent implantation after an overnight fast, 12 h after the last intake of the respective P2Y_12_ antagonist in all patients.

Exclusion criteria were a known aspirin, prasugrel, or ticagrelor intolerance (allergic reactions, gastrointestinal bleeding); a therapy with vitamin K antagonists (warfarin, phenprocoumon, acenocoumarol) or direct oral anticoagulants (rivaroxaban, apixaban, dabigatran, edoxaban); treatment with ticlopidine, dipyridamol, or nonsteroidal anti-inflammatory drugs; a family or personal history of bleeding disorders; malignant myeloproliferative disorders or heparin-induced thrombocytopenia; severe hepatic failure; known qualitative defects in platelet function; a major surgical procedure within 1 week before enrolment; a platelet count < 100.000 or > 450.000/μl; and a hematocrit < 30%.

The study protocol was approved by the Ethics Committee of the Medical University of Vienna (Ethics commitee number: 1940/2013) in accordance with the Declaration of Helsinki and its later amendments, and written informed consent was obtained from all study participants.

### Blood Sampling

All laboratory personnel were blinded to study treatment. Blood was drawn by aseptic venipuncture from an antecubital vein using a 21-gauge butterfly needle (0.8 × 19 mm; Greiner Bio-One, Kremsmünster, Austria) on day 3 after PCI. To avoid procedural deviations, all blood samples were taken by the same physician applying a light tourniquet, which was immediately released and the samples were mixed adequately by gently inverting the tubes. After the initial 3 ml of blood had been discarded to reduce peri-procedural platelet activation, blood was drawn into hirudin-coated tubes (Roche Diagnostics, Mannheim, Germany) for multiple electrode aggregometry (MEA) and into 3.8% sodium citrate Vacuette tubes (Greiner Bio-One; 9 parts of whole blood, 1 part of sodium citrate 0.129 M/L) for evaluations by flow cytometry and light transmission aggregometry (LTA).

### Determination of P-Selectin Expression and Glycoprotein (GP) IIb/IIIa Activation

**Fig. 1:**
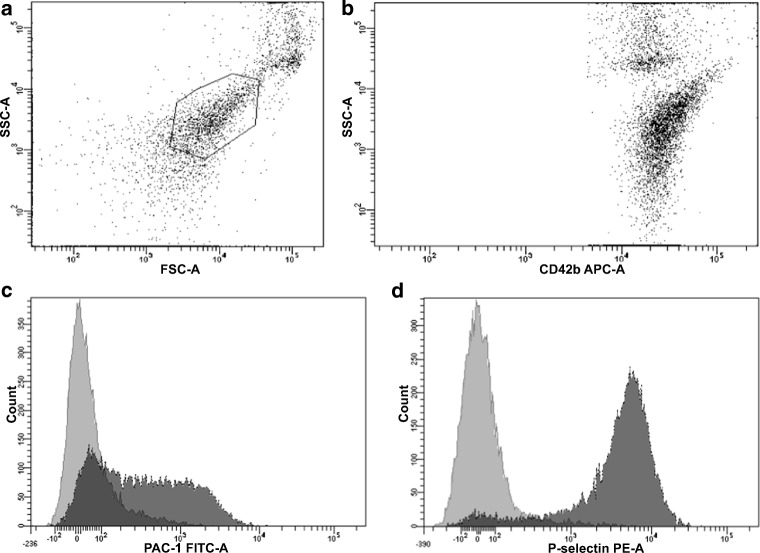
The platelet population was identified by their characteristics in the forward scatter versus side scatter plot (**a**). For analyses, this population was further identified by plotting CD42b versus side scatter (**b**). Binding of the antibodies against activated GPIIb/IIIa was determined in histograms for PAC-1 and P-selectin, respectively (**c**, **d**). The MFI for the FITC labelled PAC-1 antibody without agonist and after the addition of SFLLRN was 17 and 306, respectively. The MFI for the phycoerythrin labelled antibody against P-selectin without agonist and after the addition of SFLLRN was 0 and 4348, respectively

The expression of P-selectin and the binding of the monoclonal antibody PAC-1 to activated glycoprotein (GP) IIb/IIIa were determined in citrate-anticoagulated blood, as previously published [40, 41]. In brief, whole blood was diluted in phosphate-buffered saline to obtain 20 × 10^3^/μL platelets in 20 μL, and incubated for 10 min with the platelet-specific monoclonal antibody anti-CD42b (clone HIP1, allophycocyanin labelled; Becton Dickinson (BD), San Jose, CA, USA), without agonists, and after in vitro exposure to suboptimal concentrations of ADP (final concentration 1 μM; Roche Diagnostics GmbH, Mannheim, Germany), the TLR-1/2 agonist Pam3CSK4 (final concentration 8.9 μg/mL; InvivoGen, San Diego, USA), the TLR-4 agonist lipopolysaccharide (LPS; final concentration 1429 μg/mL; InvivoGen, San Diego, USA), the PAR-1 agonist SFLLRN (final concentration: 14.25 μM; Roche Diagnostics GmbH, Mannheim, Germany), or the PAR-4 agonist AYPGKF (final concentration 714 μM; Roche Diagnostics GmbH, Mannheim, Germany), each 10 μL for 10 min. The concentrations of all agonists were determined in previous titration experiments with increasing dosages of each agonist in 10 healthy controls. The selected concentrations of agonists induced about 60–70% of the maximal achievable increase in median fluorescence intensity (MFI) in healthy controls. Samples were then incubated for another 10 min with a mixture of antibodies against activated GPIIb/IIIa (the monoclonal antibody PAC-1-fluorescein (BD)) and P-selectin (anti-CD62p-phycoerythrin, clone CLB-Thromb6; Immuno-tech, Beckman Coulter, Fullerton, CA, USA). Isotype matched control antibodies (BD) were used for the determination of non-specific binding. After 15 min of incubation in the dark, the reaction was stopped by adding 500 μl phosphate-buffered saline (PBS) and samples were acquired immediately on a FACSCanto II flow cytometer (BD). At acquisition, the platelet population was identified by its characteristics in the forward scatter versus side scatter plot (Fig. 1a). A total of 10,000 events were acquired within this gate. This population was further identified by platelets stained with the platelet-specific monoclonal antibody anti-CD42b versus side scatter (Fig. 1b). Binding of the antibodies against activated GPIIb/IIIa and P-selectin was determined in histograms for PAC-1 and P-selectin, respectively (Fig. 1c, d). Cytometer Setup and Tracking beads (BD), which consist of FITC, PE, PERCP-CY5.5, PE-CY7, APC, APC-H7, V450, and V500-C labelled beads, were used for daily calibration of the cytometer applying the Diva software. The MFI based on all events was used for statistical calculations.

### Multiple Electrode Aggregometry

Whole blood impedance aggregometry was performed with the Multiplate analyzer (Roche Diagnostics, Mannheim, Germany) as previously described [42]. One Multiplate test cell contains 2 independent sensor units and 1 unit consists of 2 silver-coated highly conductive copper wires with a length of 3.2 mm. After dilution (1:2 with 0.9% NaCl solution) of hirudin-anticoagulated whole blood and stirring in the test cuvettes for 3 min at 37 °C, ADP (6.4 μM, Roche Diagnostics, Mannheim, Germany) or arachidonic acid (AA; final concentration of 0.5 mM; Roche Diagnostics, Mannheim, Germany) was added and aggregation was continuously recorded for 6 min. The adhesion of activated platelets to the electrodes led to an increase of impedance, which was detected for each sensor unit separately and transformed to aggregation units (AU) that were plotted against time. The AU at 6 min were used for calculations. One AU corresponds to 10 AU*min (area under the curve of AU).

### Light Transmission Aggregometry

Light transmission aggregometry (LTA) was performed on a PAP-8E aggregometer (Bio-Data, Horsham, PA USA) as previously described [42, 43]. Citrate-anticoagulated whole blood was allowed to “rest” in a tilt position at room temperature for 20 min before centrifugation. Blood tubes were centrifuged at 150×*g* for 10 min (min) at room temperature to acquire platelet-rich plasma (PRP). To obtain platelet-poor plasma (PPP), the remaining specimen were re-centrifugated at 2.000×*g* for 10 min. Platelet counts were not adjusted as the median platelet count was 198 G/L (range 166–230 G/L) [44]. The optical density of PPP was set as 100% aggregation. Platelet aggregation was initiated by addition of ADP (5 μM) or AA (1600 μM) as agonists to PRP. Optical density changes were recorded photoelectrically for 10 min as platelets began to aggregate to obtain maximal aggregation %. Maximal aggregation % was automatically calculated by the PAP-8E aggregometer by comparing the increase of light transmission through platelet-rich plasma after addition of an agonist to the baseline optical density that was set with platelet-poor plasma and considered as 100% platelet aggregation.

### Statistical Analysis

Statistical analysis was performed using the Statistical Package for Social Sciences (IBM SPSS version 25, Armonk, New York, USA). Median and interquartile range of continuous variables are shown. Categorical variables are given as number (%). We performed Mann Whitney *U* tests to detect differences in continuous variables. The chi-square test was used to assess differences in categorical variables, respectively. Two-sided *p* values < 0.05 were considered statistically significant.

## Results

Clinical and laboratory characteristics of ticagrelor (*n* = 80)- and prasugrel (*n* = 80)-treated patients are given in Table 1. The 2 groups were well-matched regarding age, sex, body mass index, hemoglobin, platelet count, renal function, comorbidities, and medication (Table 1; all *p* > 0.05). Moreover, white blood cell count (WBC), high-sensitivity C-reactive protein (hsCRP), and interleukin (IL)-6 as markers of inflammation were similar in patients with ticagrelor and prasugrel therapy, respectively (Table 1; all *p* > 0.05).

**Table 1:** Clinical and laboratory characteristics of ticagrelor- and prasugrel-treated patients

Characteristics	Ticagrelor (*n* = 80)	Prasugrel (*n* = 80)	*p*
Demographics			
Age, years	60 (51–70)	58 (51–66)	0.1
Male sex, *n* (%)	61 (76)	65 (81)	0.4
BMI, kg/m^2^	28 (25–31)	28 (25–31)	0.8
Medical history			
Previous MI, *n* (%)	13 (16)	14 (18)	0.9
Previous TIA/stroke, *n* (%)	2 (3)	3 (4)	0.7
Hypertension, *n* (%)	56 (70)	54 (68)	0.7
Hyperlipidemia, *n* (%)	58 (73)	61 (76)	0.8
Diabetes mellitus, *n* (%)	15 (19)	13 (16)	0.1
-Type I, *n* (%)	1 (1)	0 (0)	0.2
-Type II, *n* (%)	14 (18)	13 (16)	0.2
Smoking, *n* (%)	39 (49)	47 (59)	0.1
Stent implantation, *n* (%)	80 (100)	80 (100)	1
Number of stents/patient	1 (1–2)	1 (1–2)	0.3
Laboratory data			
Serum creatinine, μmol/L	88 (72–106)	83 (74–95)	0.1
Platelet count, G/L	226 (187–266)	229 (194–253)	0.9
High-sensitivity CRP, mg/L	14 (6–36)	17 (7–33)	0.5
Hemoglobin, mmol/L	8.4 (7.9–9.1)	8.6 (8.1–9)	0.7
WBC, G/L	9.3 (6.9–10.8)	8.7 (7.6–10.4)	1
Medication			
Statins, *n* (%)	79 (99)	79 (99)	1
Beta blockers, *n* (%)	78 (98)	77 (96)	0.7
ACE inhibitors, *n* (%)	61 (76)	65 (81)	0.4
Angiotensin receptor blockers, *n* (%)	19 (24)	12 (15)	0.2
Calcium channel blockers, *n* (%)	10 (13)	7 (9)	0.4

Continuous data are shown as median (interquartile range). Dichotomous data are shown as n (%)

*BMI*, body mass index; *ACE*, angiotensin converting enzyme; *CRP*, C-reactive protein; *MI*, myocardial infarction; *TIA*, transient ischemic attack; *WBC*, white blood cell count

**Fig. 2:**
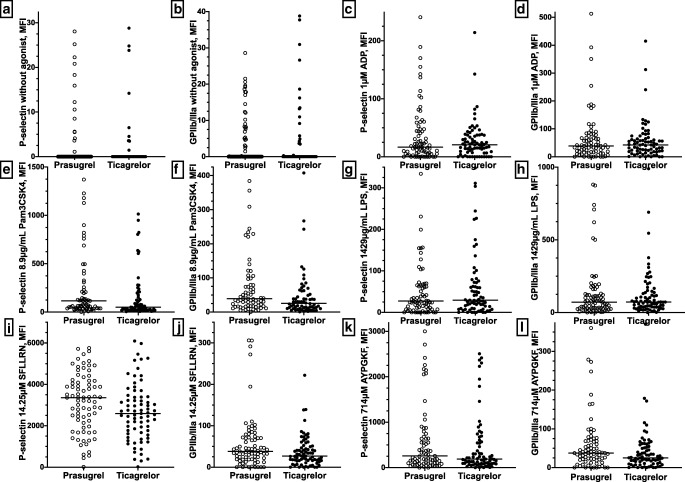
Platelet surface expression of P-selectin and activated GPIIb/IIIa in unstimulated platelets (**a** and **b**) and in response to adenosine diphosphate (ADP, [1 μM], **c** and **d**), PAM3CSK4, ([8.9 μg/mL], **e** and **f**), LPS ([1429 μg/mL], **g** and **h**), SFLLRN, ([14.25 μM], **i** and **j**) and AYPGKF, ([714 μM], **k** and **l**) in prasugrel- and ticagrelor-treated patients, respectively. The median (horizontal bar) of MFI is shown

Residual ADP-induced platelet activation as assessed by platelet surface expression of P-selectin and activated GPIIb/IIIa following in vitro exposure to ADP was very similar in ticagrelor- and prasugrel-treated patients (P-selectin ADP: 21 MFI [8–37 MFI] vs. 17 MFI [5–48 MFI], *p* = 0.9; activated GPIIb/IIIa ADP: 42 MFI [19–64 MFI] vs. 39 MFI [15–73], *p* = 0.9; Fig. 2c, d). Likewise, residual platelet aggregation in response to ADP and AA by multiple electrode aggregometry (MEA) and LTA did not differ significantly between patients receiving ticagrelor and those treated with prasugrel (Table 2; all *p* ≥ 0.3). Based on the consensus cut-off value of AU ≥ 47 by MEA [45], only 2 prasugrel-treated patients (2.5%) and no patient on ticagrelor therapy had high on-treatment residual platelet reactivity to ADP (HRPR ADP).

**Table 2:** Adenosine diphosphate (ADP)- and arachidonic acid (AA)-induced platelet aggregation by multiple electrode aggregometry (MEA) and light transmission aggregometry (LTA) in ticagrelor- and prasugrel-treated patients

	Ticagrelor (*n* = 80)	Prasugrel (*n* = 80)	*p*
MEA 6.4 μM ADP, AU	20 (15–25)	19 (15–23)	0.4
LTA 5 μM ADP, %	27 (20–33)	24 (18–33)	0.4
MEA 0.5 mM AA, AU	16 (11–20)	16 (11–21)	1
LTA 1.6 mM AA, %	3 (2–6)	2 (1–4)	0.3

Continuous data are shown as median (interquartile range)

*AA*, arachidonic acid; *ADP*, adenosine diphosphate; *AU*, aggregation units; *MEA*, multiple electrode aggregometry; *LTA*, light transmission aggregometry

TLR-1/2 mediated platelet activation as assessed by platelet surface expression of P-selectin and activated GPIIb/IIIa after stimulation with Pam3CSK4 in vitro was significantly lower in patients on ticagrelor therapy compared to those receiving prasugrel (Fig. 2e, f; Table 3; both *p* ≤ 0.005). In contrast, LPS-induced platelet surface expression of P-selectin and activated GPIIb/IIIa were similar between patients on ticagrelor and prasugrel, respectively (Fig. 2g, h, Table 3; both *p* > 0.4).

**Table 3:** Platelet surface expression of P-selectin and activated glycoprotein (GP) IIb/IIIa in response to Pam3CSK4, lipopolysaccharide (LPS), SFLLRN, and AYPGKF in ticagrelor- and prasugrel-treated patients

	Ticagrelor (*n* = 80)	Prasugrel (*n* = 80)	*p*
P- selectin expression, MFI			
Without agonist	0 (0–0.2)	0 (0–0.1)	0.4
1 μM ADP	21 (8–37)	17 (5–48)	0.9
8.9 μg/mL Pam3CSK4	51 (24–177)	116 (42–496)	0.002
1429 μg/mL LPS	30 (9–79)	28 (9–72)	0.5
14.25 μM SFLLRN	2591 (1670–3387)	3359 (2140–4170)	0.02
714 μM AYPGKF	188 (89–607)	259 (102–796)	0.2
Activated GPIIb/IIIa, MFI			
Without agonist	0 (0–0.4)	0 (0–8)	0.04
1 μM ADP	42 (19–64)	39 (15–73)	0.9
8.9 μg/mL Pam3CSK4	26 (11–54)	39 (17–107)	0.005
1429 μg/mL LPS	73 (37–146)	71 (28–127)	0.8
14.25 μM SFLLRN	27 (16–49)	38 (19–72)	0.03
714 μM AYPGKF	25 (10–52)	37 (21–72)	0.02

Continuous data are shown as median (interquartile range)

*ADP*, adenosine diphosphate; *MFI*, median fluorescence intensity; *LPS*, lipopolysaccharide

PAR-1 mediated platelet activation as assessed by platelet surface expression of P-selectin and activated GPIIb/IIIa in response to SFLLRN in vitro was significantly less pronounced in ticagrelor-treated patients compared to patients on prasugrel therapy (Fig. 2i, j, Table 3; both *p* < 0.03). Finally, PAR-4 mediated platelet activation as assessed by platelet surface expression of activated GPIIb/IIIa following stimulation with AYPGKF in vitro was significantly lower in patients receiving ticagrelor (Fig. 2l, Table 3; *p* = 0.02). AYPGKF-induced platelet surface P-selectin expression was numerically lower in patients on ticagrelor therapy compared to prasugrel-treated patients without reaching statistical significance (Fig. 2k, Table 3; *p* = 0.2).

## Discussion

Our study is the first to show that ticagrelor exerts a more pronounced antiplatelet effect on TLR-1/2 and PAR mediated platelet activation than prasugrel in ACS patients on DAPT following PCI and stenting. In contrast, ADP-induced platelet activation and aggregation, AA-induced platelet aggregation, and platelet activation via TLR-4 were similar in ticagrelor- and prasugrel-treated patients, respectively.

Previous studies have revealed that both, the PAR-1 and the PAR-4 pathway, remain active in many patients on DAPT [31, 32]. Platelet aggregation via PAR-1 and PAR-4 could even be observed in patients on prasugrel and ticagrelor, as reported recently [46].

P-selectin is stored in the α-granules of resting platelets [7]. Upon platelet activation, P-selectin is translocated to the platelet surface where it binds to its counterreceptor P-selectin glycoprotein ligand-1 on leukocytes in order to form leukocyte-platelet aggregates [47]. GPIIb/IIIa is already present on the surface of resting platelets but only converted to its active conformation following platelet activation [48]. Subsequently, activated GPIIb/IIIa serves as fibrinogen receptor thereby facilitating the interaction of platelets with one another and with other blood cells [7, 49–51]. Numerous studies have shown that both platelet-bound P-selectin and activated GPIIb/IIIa are very sensitive markers for human platelet activation [40, 52]. Furthermore, high levels of platelet surface P-selectin and activated GPIIb/IIIa in response to PAR-1 stimulation have recently been associated with an increased risk of ischemic outcomes following peripheral angioplasty and stenting [40]. Platelet aggregation in response to AA and ADP was measured in all patients by MEA and LTA. While MEA is a fast and highly standardized near point-of-care test [53], LTA was the first widely available method to assess on-treatment platelet aggregation and is still considered the historical gold standard of platelet function testing [54]. The results of both test systems have been linked to clinical outcomes in patients undergoing PCI and stenting [45, 55–57].

While ADP-induced platelet activation was very low in all patients of the study population, PAR- and TLR-mediated platelet activation was preserved in many patients. It remains to be determined if high PAR- or TLR- mediated platelet activation is associated with adverse clinical outcomes in ACS patients treated with the potent ADP P2Y_12_ antagonists. In all experiments, platelet surface expression of P-selectin was higher than that of activated GPIIb/IIIa, irrespective of the type of P2Y_12_ antagonist. This may point towards a need for higher agonist concentrations for GPIIb/IIIa activation as compared to the surface expression of P-selectin.

Although ADP-induced platelet activation and aggregation were very similar and consistently low in ticagrelor- and prasugrel-treated patients, we cannot completely exclude that a stronger inhibitory effect of ticagrelor on the P2Y_12_ receptor is responsible for the observed differences regarding TLR-1/2 and PAR-mediated platelet activation, in particular, since previous studies suggested stronger P2Y_12_ inhibition with ticagrelor compared to prasugrel [58, 59]. Given the role of ADP and the P2Y_12_ receptor for other pathways of platelet activation, stronger P2Y12 inhibition may explain the observed differences even if the ADP tests are similar as the ADP concentration in the assays may not accurately reflect the local ADP concentration at the platelet surface.

We further observed similar AA-induced platelet aggregation in patients receiving ticagrelor and prasugrel, respectively. Therefore, differences in the response to aspirin therapy did not account for the more pronounced inhibition of TLR-1/2 and PAR-mediated platelet activation by ticagrelor. Finally, patient characteristics including markers of inflammation, i.e., WBC, hsCRP and IL-6, were well-balanced between ticagrelor- and prasugrel-treated patients in our study.

Recently, Hally et al. investigated whether aspirin monotherapy or DAPT with aspirin and ticagrelor reduce platelet activation via TLR in 10 healthy individuals [60]. While aspirin alone did not affect platelet surface expression of P-selectin and activated GPIIb/IIIa in response to Pam3CSK4 and LPS, concomitant therapy with ticagrelor resulted in a modest inhibition of TLR-mediated platelet activation [60]. Furthermore, several studies found TLR and PAR signaling to be involved in the splicing of mRNA encoding for the inflammatory cytokine IL-1ß in platelets [61–63]. The observation by Jiang et al. that patients on ticagrelor therapy had significantly lower IL-1ß levels than clopidogrel-treated patients 1 year post ACS may therefore point towards inhibition of TLR and PAR mediated platelet activation by ticagrelor [64]. Together with our results, the above-mentioned findings suggest that ticagrelor at least to some extent affects platelet activation via TLR and PAR. However, whether inhibition of TLR and PAR-mediated platelet activation by ticagrelor also translates into beneficial clinical outcomes remains to be established.

Schoergenhofer et al. performed a double-blind, randomized, crossover trial with a minimum wash-out period of 6 weeks in 16 subjects to investigate whether prasugrel reduces LPS-induced coagulation activation compared to placebo [65]. They described a strong coagulation activation by LPS including increased platelet surface P-selectin expression, which was not inhibited by prasugrel, and concluded that potent P2Y_12_ inhibition does not affect coagulation activation by LPS [65]. Our results of similar and rather high levels of LPS-induced platelet surface P-selectin and activated GPIIb/IIIa in the entire study cohort suggest that (1) their findings also apply to ACS patients and (2) even ticagrelor may be unable to effectively reduce LPS-induced platelet activation in ACS.

In vivo, ticagrelor inhibits the adenosine transporter ENT1 (type 1 equilibrative nucleoside transporter) and thereby the cellular uptake of adenosine which results in higher plasma levels/ extracellular concentrations of adenosine and increased biological activity of adenosine [66]. Indeed, Nylander et al. reported that ticagrelor can augment adenosine-mediated inhibition of collagen-induced human platelet aggregation in addition to P2Y_12_ antagonism in whole blood samples from 50 healthy individuals, an effect that was not seen with the active metabolite of prasugrel in their study [36].

While prasugrel did not reduce all-cause mortality compared to clopidogrel in 13,608 ACS patients in the TRITON TIMI 38 trial [4], ticagrelor was associated with a significantly lower all-cause mortality compared to clopidogrel in 18,624 ACS patients in the PLATO study [5]. Several potential explanations for the survival benefit with ticagrelor have been discussed since publication of the PLATO results [34, 36, 67]. Given the important role of TLR and PAR in cardiovascular disease [14, 68, 69], one may speculate that the stronger inhibitory effects of ticagrelor on TLR-1/2 and PAR mediated platelet activation compared to prasugrel may at least in part contribute to the beneficial impact of ticagrelor on all-cause mortality. Indeed, in a previous study, we were able to show that high PAR-1 mediated platelet activation is a predictor of target lesion restenosis and atherothrombotic events in patients undergoing infrainguinal angioplasty and stenting [40]. However, data showing an association of PAR or TLR-mediated platelet activation with adverse outcomes in ACS patients are missing, so far. The latter is a prerequisite for future studies on therapeutic strategies in ACS patients with high platelet activation via TLR or PAR.

Selective inhibition of PAR-1 with vorapaxar on top of DAPT with aspirin and clopidogrel decreased the composite of myocardial infarction, stroke, and cardiovascular death in ACS patients in the TRACER trial, but significantly increased the risk of bleeding including intracranial hemorrhage [70]. These findings suggest that complete PAR-1 inhibition may too extensively impair cellular hemostasis, at least in conjunction with aspirin and a P2Y_12_ antagonist. Consequently, at the moment, DAPT with aspirin and a P2Y_12_ receptor antagonist remains the preferred antiplatelet regimen in ACS. New antiplatelet agents selectively targeting TLR or PAR-4 may become treatment options in ACS patients in the future [1, 71, 72].

A limitation of our study is that it was not powered for and intended to provide clinical outcome data. Moreover, the choice of the P2Y_12_ antagonist was made by the treating physician which may have led to patient selection bias. However, as shown in Table 1, the 2 study groups were well-matched regarding all relevant patient characteristics including comorbidities, laboratory markers, and concomitant medication. Accordingly, it is unlikely that differences in the patient characteristics had a significant impact on the observed results. Further, we only assessed on-treatment platelet activation in our study population, and therefore cannot comment on differences in platelet activation in response to the agonists in patients without antiplatelet therapy.

## Conclusions

Ticagrelor inhibits TLR-1/2 and PAR-mediated platelet activation in ACS patients more strongly than prasugrel. Underlying mechanisms and potential clinical implications of our observations need to be addressed in future trials.

## Abbreviations

ACS: Acute coronary syndrome
ADP: Adenosine diphosphate
DAPT: Dual antiplatelet therapy
GP: Glycoprotein
LPS: Lipopolysaccharide
LTA: Light transmission aggregometry
MEA: Multiple electrode aggregometry
MFI: Median fluorescence intensity
PAR: Protease-activated receptor
PCI: Percutaneous coronary intervention
TLR: Toll-like receptor
